# Household Food Insecurity Is Associated with Symptoms of Emotional Dysregulation in Children with Attention Deficit Hyperactivity Disorder: The MADDY Study

**DOI:** 10.3390/nu14061306

**Published:** 2022-03-20

**Authors:** Irene E. Hatsu, Leanna Eiterman, Madeline Stern, Stacy Lu, Jeanette M. Johnstone, Brenda M. Y. Leung, Priya Srikanth, Lisa Robinette, Gabriella Tost, James B. Odei, Barbara L. Gracious, L. Eugene Arnold

**Affiliations:** 1 Department of Human Sciences, The Ohio State University, Columbus, OH 43210, USA; robinette.71@buckeyemail.osu.edu; 2 OSU Extension, The Ohio State University, Columbus, OH 43210, USA; 3 Department of Neurological Surgery, The Ohio State University, Columbus, OH 43210, USA; leanna.eiterman@osumc.edu (L.E.); madeline.stern@osumc.edu (M.S.); 4 Department of Population, Family and Reproductive Health, Johns Hopkins Bloomberg School of Public Health, Baltimore, MD 21205, USA; slu47@jhmi.edu; 5 Department of Child & Adolescent Psychiatry, Oregon Health & Science University, Portland, OR 97239, USA; jojeanet@ohsu.edu (J.M.J.); tost@ohsu.edu (G.T.); 6 Helfgott Research Institute, National University of Natural Medicine, Portland, OR 97201, USA; 7 Faculty of Health Sciences, University of Lethbridge, Lethbridge, AB T1K 3M4, Canada; brenda.leung@uleth.ca; 8 School of Public Health, Oregon Health & Science University—Portland State University, Portland, OR 97239, USA; srikanth@ohsu.edu; 9 Division of Biostatistics, College of Public Health, The Ohio State University, Columbus, OH 43210, USA; odei.3@osu.edu; 10 Orange Park Medical Center, Orange Park, FL 32073, USA; barbara.gracious@hcahealthcare.com; 11 Department of Psychiatry & Behavioral Health, The Ohio State University, Columbus, OH 43210, USA; l.arnold@osumc.edu

**Keywords:** attention deficit hyperactivity disorder, food security status, emotional dysregulation, children

## Abstract

The association of household food insecurity with symptoms of attention deficit hyperactivity disorder (ADHD) and emotional dysregulation in children was examined in this study. We utilized baseline data from 134 children aged 6–12 years who were enrolled in a clinical trial investigating multinutrient supplementation as a treatment for ADHD and emotional dysregulation. Household food security status was assessed using the 18-item US Household Food Security Survey Module. The symptoms of ADHD and emotional dysregulation disorders (oppositional defiant disorder (ODD) and disruptive mood dysregulation disorder (DMDD)) were assessed using the Child and Adolescent Symptom Inventory-5 and other comorbid emotional dysregulation symptoms were assessed using the Strengths and Difficulties Questionnaire (SDQ). Multiple linear regression determined associations between household food security status and symptoms of ADHD, ODD and DMDD, emotional symptoms and conduct problems. Household food insecurity was associated with more severe emotional symptoms (β = 2.30; 95% CI = 0.87–3.73; *p* = 0.002), conduct problems (β = 1.15; 95% CI = 0.01–2.30; *p* = 0.049) and total difficulties scores (β = 4.59; 95% CI = 1.82–7.37; *p* = 0.001) after adjusting for covariates (child’s sex, parent marital status, household income, parental anxiety and other parental psychopathology). In unadjusted analyses, household food insecurity was also associated with increased ODD (β = 0.58; 95% CI = 0.21–0.95; *p* = 0.003) and DMDD symptoms (β = 0.69; 95% CI = 0.20–1.19; *p* = 0.006), but these associations attenuated to non-significance after adjusting for all covariates. Household food insecurity was associated with more severe emotional dysregulation symptoms. Discussing and addressing food insecurity may be appropriate initial steps for youths with ADHD and emotional dysregulation.

## 1. Introduction

Attention deficit hyperactivity disorder (ADHD) is a common neuropsychiatric disorder that is usually diagnosed in childhood and often has long-lasting impacts in adolescence and adulthood [1]. Characterized by symptoms of inattention, hyperactivity and impulsivity that impair functionality, ADHD is frequently comorbid with other neurodevelopmental and psychiatric disorders. ADHD has global community prevalence estimates of 2–7%, with 50–90% of those experiencing additional psychiatric diagnoses [2,3]. Emotional dysregulation symptoms of irritability, anger and aggression commonly co-occur with ADHD [4]. More severe and impairing symptoms of emotional dysregulation may warrant an additional diagnosis of oppositional defiant disorder (ODD) or disruptive mood dysregulation disorder (DMDD). ODD is characterized by a pattern of frequent engagement in disruptive interpersonal behavior (such as defiance), whereas DMDD is characterized by frequent and severe temper outbursts [5]. The severity and frequency of emotional and physical outbursts are higher in children with DMDD than in children without DMDD [5].

The exact etiologies of ADHD, ODD and DMDD remain uncertain. However, they appear to result from the interaction of genetic, environmental, biological and psychosocial factors that impact neurobiological development, which is similar to other mental disorders in youth [6]. ADHD is established as 70% heritable and a component of this genetic diathesis may be an increased vulnerability to poor diet and other environmental stressors [7]. Several dietary factors, including micronutrient status, have been associated with ADHD and ODD symptoms [8]. It is not clear whether the same is true for DMDD, which is a new addition to the DSM-5, as risk factors are still being explored within research [9]. A large extent of diet risk evidence for ADHD is focused on processed foods [10], deficiencies in specific nutrients [11] and dietary additives [12]. The contributions of food intake and dietary characteristics remain an area of growing interest in the etiology and management of ADHD, as with other psychiatric disorders [13].

Food insecurity is a global public health concern due to the consequences of poor dietary outcomes across a lifespan. It is defined as limited or uncertain food availability or the inability to acquire nutritionally adequate and safe foods in socially acceptable ways [14]. In 2019, approximately 10.7 million children in US households (13.6%) [15] and 1.2 million children in Canadian households (17.3%) [16] were exposed to food insecurity, which increases their risk of inadequate nutrient intake, anemia, asthma and other poor physical health outcomes [17]. Childhood food insecurity has also been associated with cognitive, emotional and behavioral problems in children [17]. Specifically, food insecure children tend to have increased levels of aggression, anxiety, depression and suicidal ideation, as well as hyperactivity and inattention [18]. 

A recent review of the literature on the association between food insecurity and ADHD symptoms in children highlighted the need for more research in this area [19]. Given the high global prevalence of childhood ADHD and the comorbidity of emotional and behavioral problems in children with ADHD [4], an adequate evidence base is needed to establish childhood food insecurity as an environmental risk factor. This study builds on prior knowledge by assessing the relationships between household food security status and symptom severity in a pediatric population with symptoms of ADHD and emotional dysregulation.

## 2. Materials and Methods

### 2.1. Study Design and Participants

This study is a cross-sectional secondary analysis of baseline data from children participating in the Micronutrients for ADHD in Youth (MADDY) study. The MADDY study is a multisite clinical trial comparing the efficacy of a broad-spectrum nutrient supplement to a placebo as a treatment for the symptoms of ADHD and co-occurring irritability [20,21]. Participants included in the study were aged 6–12 years and met the following Diagnostic and Statistical Manual for Mental Disorders (DSM-5) criteria for ADHD: a clinical cut-off of six or more inattentive or hyperactive/impulsive symptoms endorsed via parent report on the ADHD subscales of the Child and Adolescent Symptom Inventory-5 (CASI-5) [22]. These symptoms had to be present in more than one setting and cause impairment in social or academic functioning or both. In addition, participants had to have had at least one symptom of irritability or anger, as assessed by the ODD and DMDD subscales from the CASI-5. Study participants were recruited through referrals from pediatricians, mental health care providers and social media platforms at the three study sites (Columbus, OH, and Portland, OR, in the USA and Lethbridge, AB, in Canada) as well as affiliated child psychiatry divisions and children’s hospitals at the two US sites. Written informed consent from parents and child assent were obtained using forms and procedures approved by each site’s Institutional Review Board.

### 2.2. Measures

Study questionnaires were self-administered (by a parent or care giver) and completed in person through the web-based data management platform REDCap. This allowed for accurate and efficient data collection across all study sites. Structured questionnaires were used to assess child, parent and household characteristics. Items assessed included participant age, sex, race, parents’ level of education and parents’ mental health status, as well as household income and size. 

The validated 18-item US Household Food Security Survey Module was used to assess food security status [23]. Participant responses were scored following the standard administration procedures and were used to classify study participants’ households into four categories: (1) high food security (i.e., no affirmative response on the survey, which indicates no food access problems for family members); (2) marginal food security (i.e., 1–2 affirmative responses, which indicates some anxiety about food adequacy but no changes to food intake); (3) low food security (i.e., 3–7 affirmative responses, which indicates some reduction in diet quality but not in food intake); and (4) very low food security (i.e., eight or more affirmative responses, which indicates reduced food intake and disrupted eating patterns) [23]. Due to the overall sample size, the first two categories were further collapsed into the food secure (FS) group, while the latter two were combined into the food insecure (FI) group [24]. 

ADHD symptoms of inattention and hyperactivity/impulsivity, as well as comorbid emotional dysregulation symptoms of ODD and DMDD, were evaluated using the CASI-5 [22]. The CASI-5 is a behavior rating scale that has been validated for assessing mental health conditions in children aged 5–18 through parent and teacher ratings. Its subscales correspond to the symptom criteria defined by the DSM-5. The parent versions of the subscales were used in this study to capture the symptoms of ADHD, DMDD and ODD using the questions from the corresponding subscales. Symptom counts in each subscale indicated whether the child had the number of symptoms consistent with a DSM-5 diagnosis. The items in each subscale were rated on a frequency of occurrence scale from 0 (never) to 3 (very often) and then summed to determine the severity scores for each disorder [22]. The range of symptom severity scores for each symptom was as follows: 0 to 27 for inattention; 0 to 27 for hyperactivity/impulsivity; 0 to 24 for ODD; and 0 to 6 for DMDD [22].

In addition to ODD and DMDD, the emotional symptoms and conduct problems subscales of the Strengths and Difficulties Questionnaire (SDQ) were used to measure emotional dysregulation, as well as the total difficulties scores. The SDQ is a 25-item (five scales of five items) questionnaire that has been shown to accurately identify child and adolescent emotional and behavioral problems [25]. Parent reports on the scale items were scored as 0 (not true), 1 (somewhat true) and 2 (certainly true), with a summed score range of 0–10 for each subscale and 0–40 for the total difficulties score (a sum of the scores from four of the five subscales). The recommended cut-off for each scale (described below) was applied to categorize the scores as “normal”, “borderline” and “abnormal” difficulties [26].

### 2.3. Statistical Analysis

Descriptive statistics were used to compare child and parent characteristics according to food security status. The item mean score for each CASI-5 subscale was calculated to provide clinical interpretation. The CASI-5 item mean score was further dichotomized into ≥2 and <2, where ≥2 was considered clinically impairing or borderline–abnormal. For the SDQ subscales, cut points of greater than or equal to 3, 4 and 14 were used to dichotomize conduct problems, emotional problems and total difficulties scores, respectively. Scores greater than or equal to these thresholds were considered clinically impairing or borderline–abnormal [26].

Either a two-sample *t*-test with equal or unequal variances (when the ratio of the two standard deviations was >2) was used to examine differences in the continuous variables between food secure and food insecure households or a Mann–Whitney test was used when the distribution was not symmetric. Pearson’s chi-squared or Fisher’s exact test (when expected cell counts were <5) were used for the categorical variables.

Linear regression models were used to examine mean differences in the CASI-5 subscales and SDQ subscales for symptoms of ADHD and mood dysregulation between those who experienced food insecurity and those with food security. Beta coefficients (β) and corresponding 95% confidence intervals (95% CI) are reported to quantify the mean differences in CASI-5 and SDQ subscales for households experiencing food insecurity compared to households with food security. The base model is the unadjusted association (Model 0). We assessed for the potential confounding of other covariates by adding one variable at a time to the base model. When the beta coefficient for food insecurity changed by ≥10% with the addition of a covariate, we then considered that covariate to be a potential confounder and added it to the model. We assessed for multicollinearity using a variance inflation factor cut-off of ≥5. The final model consisted of covariates that were associated with food insecurity status, clinically meaningful and/or considered to be potential confounders. All analyses were conducted using SAS software 9.4 (SAS Institute, Cary, NC, USA) and STATA (Release 16, College Station, TX, USA). Statistical significance was defined as two-sided: *p*-value < 0.05.

## 3. Results

The overall characteristics of the children and parents in the cohort are presented in Table 1, stratified by food security status. Of the 134 children included in the study, ~72% were male and about 82% identified as white. The mean age of participants was 9.31 (SD = 1.69) years and more than half (55%) lived in households with an annual income of over $80,000. Overall, 10.4% (8.4% in the US sample and 15.9% in the Canadian sample) of the study participants lived in food insecure households. Specifically, 7.5% lived in households with low food security and 3.0% were from households experiencing very low food security. Due to the small size of the food insecure sample, the remainder of the findings are reported for the overall sample and not separated by country. Food security status was significantly associated with household income, parent education and parental anxiety, as well as parental non-anxiety and non-mood psychopathology (all *p* < 0.05) (Table 1).

Household income was inversely associated with the severity of hyperactivity/impulsivity symptoms (β = −0.25; *p* = 0.003), DMDD (β = −0.19; *p* = 0.027), ODD (β = −0.29; *p* < 0.001), emotional symptoms (β = −0.35; *p* < 0.001), conduct problems (β = −0.31; *p* < 0.001) and total difficulties score (β = −0.43; *p* < 0.001). Similarly, parental anxiety and other non-anxiety and non-mood psychopathology were associated with the severity of child ODD, emotional symptoms, conduct problems and total difficulties score (all *p* < 0.01). Parental psychopathology other than anxiety and mood were also associated with severe child DMDD symptoms (β = 0.23; *p* < 0.007).

A significantly higher proportion of children from food insecure households had clinically impairing symptoms compared to those in food secure households. As shown in Table 2, among the children from food insecure households, 100% had clinically impairing symptoms of inattention (*n* = 14), 71% had clinically impairing ODD or DMDD (*n* = 10) and 93% had borderline–abnormal levels of emotional symptoms (*n* = 13). Children who lived in food insecure households had significantly higher mean inattention and emotional dysregulation symptom scores compared to those who lived in food secure households (all *p*-values < 0.05, except inattention and hyperactivity/impulsivity) (Table 3).

Our total sample size for regression analyses was 132, with *n* = 118 in the food secure group and *n* = 14 in the food insecure group, because two responses were missing data for parental anxiety and other parental behavioral problems. In the unadjusted regression analyses (Model 0, Table 4), increases of 0.58 (95% CI = 0.21–0.95), 0.69 (95% CI = 0.20–1.19), 2.81 (95% CI = 156–4.06), 2.22 (95% CI = 1.22–3.22) and 6.76 (95% CI = 4.25–9.26) were observed in the item means of ODD and DMDD, as well as in the scores for emotional symptoms, conduct problems and total difficulties, respectively, among children in food insecure households compared to those in food secure households. The associations remained after adjusting for the child’s sex (Model 1, Table 4). Our final model (Model 2) included the covariates of the child’s sex, parent marital status, household income, parental anxiety and other parental non-anxiety and non-mood psychopathology. We did not include parental education status in our final model because of its multicollinearity with household income. The Model 1 associations attenuated to non-significance for ODD and DMDD after additionally adjusting for household income, parental anxiety and other parental psychopathology, which were all associated with food insecurity status. The associations for emotional symptoms (β = 2.30; 95% CI = 0.87–3.73; *p* = 0.002), conduct problems (β = 1.15; 95% CI = 0.01–2.30; *p* = 0.049) and total difficulties score (β = 4.59; 95% CI = 1.82–7.37; *p* = 0.001) remained significant even after adjusting for all covariates (Model 2, Table 4).

## 4. Discussion

Food insecurity and neurodevelopmental disorders, such as ADHD, both present significant public health problems. We sought to determine the relationship between household food security status and the symptoms of emotional dysregulation within a pediatric population with ADHD symptoms that was taking part in a clinical trial for micronutrient supplementation. Approximately 10.4% of the study cohort lived in food insecure households in which there was uncertainty around the ability to acquire adequate nutritious food for family members. This prevalence was slightly lower than the national averages for food insecure households with children in both the US (13.6%) and Canada (17.3%) [15,16], possibly because of this sample’s bias towards higher SES, especially higher income.

This study showed that household food insecurity was associated with more severe emotional and conduct problems. Household food insecurity was also associated with more severe ODD and DMDD symptoms in the unadjusted and sex-adjusted models. These findings add to and extend the existing literature about diet-related influences on the comorbid emotional dysregulation symptoms that are experienced by children with ADHD symptoms. The findings also support previous research that food insecurity worsens emotional problems, with possible lasting impacts in adolescence and adulthood [18]. A plausible mechanism by which food insecurity could elicit emotional dysregulation symptoms is inadequate diet and nutrient intake. Food insecure households are at an increased risk of poor dietary intake and patterns due to likely constraints in food quantity and/or quality [27]. Micronutrient insufficiency or deficiency could result from the consumption of energy-dense but nutrient-poor foods, which are prioritized in food insecure households due to their low cost. Vitamins and minerals, as well as omega-3 fatty acids, are essential to the cascade of brain actions that support emotional and behavioral regulation: processes that may be impacted if nutrients are deficient [28,29]. Another plausible mechanism is poor parental wellbeing. Food insecurity is thought to increase adult stress levels, parental distress and other mental health challenges, which, in turn, impairs the quality of parenting, family environmental effects and child outcomes [30,31]. Therefore, the members of families living with food insecurity may experience and express more negative emotions. 

All of the children in food insecure households experienced clinically impairing symptoms of inattention. However, in contrast to prior studies that examined the relationship between household food insecurity and ADHD symptom severity in age groups similar to ours, household food insecurity was not independently associated with inattention or with hyperactivity/impulsivity in this sample [32,33]. A plausible explanation for the difference in findings may be the use of different tools in assessing both household food insecurity and ADHD symptoms. None of the prior studies utilized CASI-5 while using DSM-5 criteria to assess symptoms. Moreover, most studies also used non-standard or older versions of the 18-item Household Food Security Survey Module compared to that used in this study [18]. The size of the food insecure sample could have also contributed to the difference in findings. Although the parent study (i.e., the MADDY clinical trial) is adequately powered to detect differences between two groups of nearly equal size (60:40 split) in a sample of 134, the relatively smaller size of the food insecure sample may have decreased power for our purposes. While very low food security has been associated with inattention and hyperactivity, only five of the study participants lived in households that experienced this level of food insecurity. Nevertheless, it is interesting that the ADHD symptom cluster that came closest to attaining significance was hyperactivity/impulsivity, which is more closely related to emotional instability than inattention.

This study’s strengths include having participants from three different areas: two in the United States and one in Canada. The homogenous nature of our sample is acknowledged as a limitation; however, this is consistent with racial and ethnic disparities in ADHD diagnosis among children [34]. Another limitation is the relatively small sample size of food insecure families, which makes it difficult to fully analyze all of the covariates and to generalize our findings. For example, our study did not account for dietary and nutrient intake, which is affected by household food insecurity. One difficulty in teasing out the contribution of food insecurity to emotional dysregulation is the collinearity of family income and parent education with both child clinical symptoms and food insecurity. While we excluded parent education, we did adjust for family income. This could account for the attenuation of the magnitude of association when those covariates were added to the models. Nevertheless, some clinical associations with food insecurity remained even after adjusting for family income. In the future, path analyses or longitudinal studies may provide a clearer picture of whether food insecurity is mainly a marker for the other risk factors, a mediator of their effects or a result of an independent risk.

## 5. Conclusions

This study provides evidence of a direct relationship between exposure to household food insecurity and the severity of emotional dysregulation symptoms among children who have ADHD. Food insecurity has independently been associated with child emotional development. The current findings support the need to routinely screen for food insecurity when assessing pediatric behavioral and emotional symptoms [19]. This aligns with the recommendations of the American Academy of Pediatrics for the regular screening of children for exposure to food insecurity within the clinical setting. This practice helps to identify food insecure children who could benefit from targeted nutritional resources and interventions to improve their health, such as referrals to federal nutrition assistance programs or local food pantries and banks. Our findings contribute to the growing scientific evidence of the effect of food insecurity on pediatric mental health. The replication of this work with a larger sample, oversampled for children in food insecure households, is needed to confirm the primary relationships found in the present study. Longitudinal studies are also needed to further elucidate the role of food insecurity in the etiology and course of ADHD, DMDD, ODD and other child psychiatric disorders.

## Figures and Tables

**Table 1:** Participant characteristics according to household food security status.

	Total	Food Secure	Food Insecure	*p*-Value
	(*n* = 134)	(*n* = 120)	(*n* = 14)	
**Child Characteristics**	*n* (%)			
Age, Mean (SD)	9.31 (1.69)	9.31 (1.73)	9.36 (1.34)	0.919
Sex				0.519
Male	96 (71.64)	87 (72.50)	9 (64.29)	
Female	38 (28.36)	33 (27.50)	5 (35.71)	
Race				0.683 ^a^
American Indian/Alaskan Native	0 (0.00)	0 (0.00)	0 (0.00)	
Asian	3 (2.24)	3 (2.50)	0 (0.00)	
African American	4 (2.99)	3 (2.50)	1 (7.14)	
Native Hawaiian/Pacific Islander	1 (0.75)	1 (0.83)	0 (0.00)	
White	110 (82.09)	99 (82.50)	11 (78.57)	
Other	7 (5.22)	6 (5.00)	1 (7.14)	
Decline to answer	9 (6.72)	8 (6.67)	1 (7.14)	
**Parent/Household Characteristics**	*n* (%)			
Household income				<0.001
≤$30,000	13 (9.70)	8 (6.72)	5 (33.33)	
$30,001–$60,000	29 (21.64)	22 (18.49)	7 (46.67)	
$60,001–80,000	17 (12.69)	16 (13.45)	1 (6.67)	
>$80,001	74 (55.22)	72 (60.50)	2 (13.33)	
Do not know	1 (0.75)	1 (0.84)	0 (0.00)	
Household size, Mean (SD)	4.51(1.24)	4.47 (1.19)	4.86 (1.61)	0.268
Parent Education				0.015
≤High School	18 (13.43)	17 (14.17)	1 (7.14)	
Tech/Professional College	35 (26.12)	26 (21.67)	9 (64.29)	
≥University Degree	79 (58.96)	75 (62.50)	4 (28.57)	
Other	2 (1.49)	2 (1.67)	0 (0.00)	
Parent Marital Status				0.158
Single	11 (8.21)	8 (6.67)	3 (21.43)	
Married	97 (72.39)	90 (75.00)	7 (50.00)	
Divorced	18 (13.43)	15 (12.50)	3 (21.43)	
Common Law	8 (5.97)	7 (5.83)	1 (7.14)	
Parental ADHD diagnosis				0.906
Yes	69 (51.49)	62 (51.67)	7 (50.00)	
No	65 (48.51)	58 (48.33)	7 (50.00)	
Parental Depression ^b^				0.73 ^a^
Yes	25 (18.94)	22 (18.64)	3 (21.43)	
No	107 (81.06)	96 (81.36)	11 (78.57)	
Parental Anxiety ^b^				0.008
Yes	51 (38.64)	41 (34.75)	10 (71.43)	
No	81 (61.36)	77 (65.25)	4 (28.57)	
Parental Manic Depression ^b^				0.20 ^a^
Yes	8 (6.06)	6 (5.08)	2 (14.29)	
No	124 (93.94)	112 (94.92)	12 (85.71)	
Other parental psychopathology ^b^				0.001
Yes	48 (36.36)	37 (31.36)	11 (78.57)	
No	84 (63.64)	81 (68.64)	3 (21.43)	

^a^ Fisher’s exact test; ^b^ two missing parental anxiety, parental depression, parental manic depression and other parental psychopathology responses.

**Table 2:** The proportion of participants with severe symptoms according to household food security status.

Symptom Severity Classification	Food Secure	Food Insecure	*p*-Value ^a^
	(*n* = 120)	(*n* = 14)	
**CASI-5**	*n* (%)		
Inattention			0.01
Not clinically impairing	38 (31.67)	0 (0)	
Clinically impairing	82 (68.33)	14 (100.00)	
Hyperactivity/Impulsivity			0.151
Not clinically impairing	54 (45.00)	3 (21.43)	
Clinically impairing	66 (55.00)	11 (78.57)	
ODD			0.017
Not clinically impairing	78 (65.00)	4 (28.57)	
Clinically impairing	42 (35.00)	10 (71.43)	
DMDD			0.005
Not clinically impairing	84 (70.00)	4 (28.57)	
Clinically impairing	36 (30.00)	10 (71.43)	
**Strength and Difficulties Questionnaire**	*n* (%)		
Emotional symptoms			<0.001
Normal	69 (57.50)	1 (7.14)	
Borderline–Abnormal	51 (42.50)	13 (92.86)	
Conduct problems			0.066
Normal	38 (31.67)	1 (7.14)	
Borderline–Abnormal	82 (68.33)	13 (92.86)	
Total Difficulties			0.072
Normal	25 (20.83)	0 (0.00)	
Borderline–Abnormal	95 (79.17)	14 (100.00)	

^a^ Fisher’s exact test; ADHD, attention deficit hyperactivity disorder; ODD, oppositional defiant disorder; DMDD, disruptive mood dysregulation disorder.

**Table 3:** Differences in ADHD and emotional dysregulation symptom severity according to household food security status.

Symptoms (Observed Range)	Total (*n* = 134) Mean (SD)	Food Secure (*n* = 120) Mean (SD)	Food Insecure (*n* = 14) Mean (SD)	*p*-Value ^a^
ADHD Inattention (1–3)	2.22 (0.48)	2.21 (0.50)	2.35 (0.28)	0.301
ADHD Hyperactivity/Impulsivity (0.33–3)	1.97 (0.74)	1.93 (0.76)	2.33 (0.50)	0.053
ODD (0.13–3)	1.70 (0.68)	1.64 (0.67)	2.21 (0.59)	0.003
DMDD (0–3)	1.38 (0.89)	1.30 (0.86)	2.00 (1.00)	0.005
Emotional Symptoms (0–10)	3.40 (2.38)	3.10 (2.28)	5.93 (1.69)	<0.001
Conduct Problems (0–10)	3.65 (1.90)	3.42 (1.73)	5.64 (2.17)	<0.001
Total Difficulties (7–35)	18.09 (4.94)	17.38 (4.34)	24.21 (5.66)	<0.001

^a^*p*-value from *t*-test; ADHD, attention deficit hyperactivity disorder; ODD, oppositional defiant disorder; DMDD, disruptive mood dysregulation disorder.

**Table 4:** The associations of food security status with attention deficit hyperactivity disorder and mood dysregulation symptoms.

	Model 0	Model 1	Model 2
	β (95% CI)		
ADHD Inattention	0.14	0.15	0.21
	(−0.13, 0.41)	(−0.12, 0.42)	(−0.12, 0.55)
ADHD Hyperactivity/Impulsivity	0.4	0.41 *	0.23
	(−0.01, 0.80)	(0.01, 0.82)	(−0.25, 0.72)
ODD	0.58 **	0.59 **	0.27
	(0.21, 0.95)	(0.22, 0.96)	(−0.16, 0.69)
DMDD	0.69 **	0.71 **	0.5
	(0.20, 1.19)	(0.22, 1.20)	(−0.08, 1.08)
Emotional Symptoms	2.81 ***	2.82 ***	2.30 **
	(1.56, 4.06)	(1.56, 4.08)	(0.87, 3.73)
Conduct Problems	2.22 ***	2.24 ***	1.15 *
	(1.22, 3.22)	(1.24, 3.24)	(0.01, 2.30)
SDQ Total Difficulties	6.76 ***	6.79 ***	4.59 ***
	(4.25, 9.26)	(4.28, 9.31)	(1.82, 7.37)

ADHD, attention deficit hyperactivity disorder; ODD, oppositional defiant disorder; DMDD, disruptive mood dysregulation disorder; Model 0, unadjusted; Model 1, Model 0 + sex; Model 2, Model 1 + family income + marital status + parental anxiety + other parental psychopathology; β, beta coefficient; CI, confidence interval; *** *p* < 0.001; ** *p* < 0.01; * *p* < 0.05.

## Data Availability

The dataset for this manuscript is not currently available because the data are part of a clinical trial whose findings are still being analyzed for dissemination. The data will be made available after the data analysis and the publication of the main outcome papers.
